# The interaction of lipids and inflammatory markers predict negative symptom severity in patients with schizophrenia

**DOI:** 10.1038/s41537-021-00179-8

**Published:** 2021-10-20

**Authors:** David R. Goldsmith, Nicholas Massa, Brian J. Miller, Andrew H. Miller, Erica Duncan

**Affiliations:** 1 Emory University School of Medicine, Department of Psychiatry and Behavioral Sciences, Atlanta, GA USA; 2 Atlanta Veterans Affairs Health Care System, Decatur, GA USA; 3 Augusta University, Department of Psychiatry and Health Behavior, Augusta, GA USA

**Keywords:** Schizophrenia, Biomarkers, Schizophrenia

## Abstract

Finding biological predictors and novel mechanisms underlying negative symptoms of schizophrenia is of significant importance given the lack of effective treatments. Increasing data support a role for metabolic dysfunction and inflammation in reward processing deficits in psychiatric illness. Herein, we found an interaction between lipids and inflammation as a predictor of worse negative symptom severity in individuals with schizophrenia. Future studies may seek to further elucidate this relationship and thereby reveal novel treatment targets for negative symptoms.

There is increased focus on the interaction of inflammatory and metabolic pathways in psychiatric disorders^1–4^. Individuals with schizophrenia consistently show disruptions of several metabolic parameters, including lipid and glucose homeostasis, which may be associated with underlying genetic risk for the disorder^5,6^. Similarly, many individuals with schizophrenia have increased inflammatory markers^7^, which have previously been associated with negative symptom severity^8^.

Both systemic inflammation and metabolic disturbances have effects on the brain^9,10^. Our group has previously shown that peripheral biomarkers and gene transcripts related to glucose and lipid metabolism predicted response to the anti-inflammatory effects of infliximab in patients with depression^4,11^. We have also recently demonstrated that markers of altered glucose metabolism were associated with decreased functional connectivity in reward circuitry, an effect that was only seen in individuals with increased inflammation in patients with depression^2^.

These relationships have not been investigated relative to negative symptoms of schizophrenia, which are also marked by alterations in reward processing^12^. Accordingly, we examined negative symptoms and non-fasting levels of plasma lipid markers as well as plasma concentrations of two of the most commonly altered cytokines in patients with schizophrenia: tumor necrosis factor (TNF) and interleukin-6 (IL-6), which have both been shown to be associated with negative symptoms^13,14^. We also repeated the analyses in a second independent dataset in order to replicate these findings.

Sociodemographic and clinical variables as well as immune and metabolic marker concentrations for the primary and replication sample are shown in Table 1.

**Table 1:** Sociodemographic and clinical variables as well as immune and metabolic marker concentrations in the primary sample (*n* = 52 patients) and the replication sample (*n* = 65).

	Primary sample	Replication sample
Age	51.3 (9.2)	41.69 (12.4)
Sex	49/52 male (94.2%)	34/65 male (52.3%)
Race	39/52 black (75%)	46/65 black (70.8%)
Smoking	26/52 smoker (50%)	8 cigarettes smoked daily (9.5)
BMI	31.4 (6.8)	32.2 (7.7)
PANSS total score	60.6 (14.5)	70.9 (16.7)
PANSS positive subscale score	15.8 (4.8)	18.8 (6.4)
PANSS negative subscale score	16.6 (6.0)	16.1 (5.8)
PANSS general subscale score	28.2 (7.8)	36.0 (9.6)
Total cholesterol	176 (120–289)	161 (86–283)
LDL	111 (46.8–195)	89 (20–181)
ln TNF (mean-centered)	−0.32 (−1.53 to 3.24)	1.78 (−1.90 to 3.69)
ln IL-6 (mean-centered)	−0.21 (−2.67 to 3.88)	1.62 (0.29 to 6.63)

All variables are shown as means and standard deviations or proportions, except for TNF, IL-6, cholesterol, LDL, which are shown as median and min/max.

*BMI* body mass index, *PANSS* positive and negative syndrome scale, *LDL* low-density lipoprotein, *ln TNF* natural log of tumor necrosis factor, *ln IL-6* natural log of interleukin-6.

Of the metabolic markers, only total cholesterol (*r* = 0.387, *p* = 0.005) and LDL (*r* = 0.356, *p* = 0.009) were significantly correlated with the PANSS negative subscale score (Fig. 1). No metabolic markers were correlated with total PANSS score or PANSS positive or general scores. As such, only total cholesterol and LDL, as well as PANSS negative subscale score, were used in subsequent analyses. Cholesterol and LDL had evidence of increased kurtosis/skewness, and as such both values were log-transformed and mean-centered for use in all further analyses. Neither TNF, IL-6, or their combination were significantly correlated with total, positive, negative, and general PANSS scores or individual subscale scores.

**Fig. 1:**
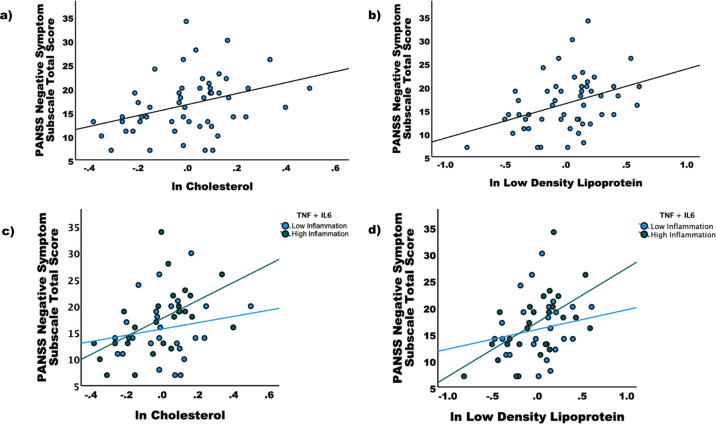
Correlations between Positive and Negative Syndrome Scale (PANSS) Negative Symptom Subscale Score and total cholesterol and low-density lipoprotein (LDL). Of the metabolic markers, only (**a**) total cholesterol (*r* = 0.387, *p* = 0.005) and (**b**) LDL (*r* = 0.356, *p* = 0.009) were significantly correlated with the PANSS negative subscale score. The sample was split between those with high and low inflammation as indexed by a median split of a composite score of tumor necrosis factor (TNF) and interleukin-6 (IL-6). In the low inflammation subgroup (**c**), there was no relationship between PANSS Negative Symptom Subscale Score and cholesterol (*p* = 0.514) or LDL (*p* = 0.317), whereas in the high inflammation subgroup (**d**) significant correlations were found for total cholesterol (*p* = 0.001) and LDL (*p* = 0.002).

The sample was split into a high and low inflammation group based on a median split of the *z*-scored combination of TNF and IL-6. There was no relationship between PANSS negative symptoms and either cholesterol (*r* = 0.134, *p* = 0.514) or LDL (*r* = 0.204, *p* = 0.317) in the low inflammation group. In the high inflammation group, there was a significant relationship between PANSS negative symptoms and both cholesterol (*r* = 0.599, *p* = 0.001) and LDL (*r* = 0.582, *p* = 0.002) (Fig. 1).

In order to test the interaction effect between inflammatory and metabolic markers, we conducted linear regression models including cytokine group, metabolic marker group, and their interaction in addition to relevant covariates. There was a significant interaction between the TNF group and the LDL group for negative symptoms (*p* < 0.05), but not for the other markers. The TNF + IL-6 group × LDL group significantly predicted negative symptoms (β = 1.533, *p* = 0.016) as well as individual negative symptom items including blunted affect (*r* = 0.293, *p* = 0.035) and emotional withdrawal (*r* = 0.346, *p* = 0.012).

In the replication sample, the TNF + IL-6 group × cholesterol group interaction correlated with emotional withdrawal (*r* = 0.280, *p* = 0.030) as did the TNF + IL-6 group × LDL group interaction (*r* = 0.280, *p* = 0.030), controlling for age, sex, race, smoking, and BMI (see Supplementary Materials for more detail).

This study demonstrated interactive relationships between lipid metabolism and inflammatory markers predicting negative symptom severity in a sample of patients with schizophrenia. We were also able to replicate findings in a second, independent dataset. Though we have previously shown relationships between increased inflammation and negative symptoms of schizophrenia, this is the first study to support a role for a relationship between negative symptoms and lipid metabolism in patients with schizophrenia that is dependent on inflammation. This is consistent with recent evidence that high triglycerides and high inflammation together predicted negative symptom severity and worse outcomes at 1-year follow-up in first episode psychosis^3^. Obesity and metabolic dysfunction drive systemic inflammation via the activation of macrophages in adipose tissue^15^. Inflammatory cytokines released by macrophages play a critical role in the development of hyperlipidemia and insulin resistance, which in turn, leads to metabolic disturbances that further promote cytokine production from adipose tissue, thus contributing to chronic inflammation^16^. Though this study was limited by small sample sizes, future work should seek to further investigate the bidirectional impact of inflammation and metabolic dysfunction as a driver of negative symptom severity in patients with schizophrenia as they could represent potential targets for novel therapeutics.

## Methods

Fifty-two chronic outpatients with a primary diagnosis of schizophrenia or schizoaffective disorder were recruited from the Atlanta Veterans Affairs Health Care System (Atlanta VAHCS) as part of two different parent studies. All subjects provided written informed consent for the study was approved by the Emory University Institutional Review Board and the Atlanta VAHCS Research and Development Committee. Inclusion/exclusion criteria as well as descriptions of the inflammatory markers have been previously described (see Supplementary Materials)^17^. Blood samples for inflammatory markers were obtained in chilled EDTA-coated tubes and spun at 2000 × *g* for 15 min at 4 °C, and plasma was collected and stored at −80 °C for later batched analysis. Non-fasting lipid panels [total cholesterol, low-density lipoprotein (LDL), high-density lipoprotein (HDL), very low-density lipoprotein (VLDL), and triglycerides] were obtained from the electronic medical record for the date closest to the study visit (blood sampling and clinical interview). Negative symptoms, both total score and individual items were assessed using the Positive and Negative Syndrome Scale (PANSS).

Inflammatory markers were not normally distributed and were thus log-transformed. Moreover, as the inflammatory markers were run at different times, the concentrations were mean-centered (the batch effect was accounted for as a covariate in analyses). Median splits for both the metabolic and inflammatory markers were calculated in order to determine high and low groups. Linear regression models were used to determine the relationship between the interaction of metabolic and inflammatory markers with negative symptom severity including the following covariates: age, sex, race, smoking, body mass index (BMI), batch effect, and time between blood samples for metabolic and inflammatory markers. A replication sample including 58 individuals with schizophrenia was used to replicate the findings from the primary sample (see Supplementary Materials)^14^.

### Reporting Summary

Further information on research design is available in the Nature Research Reporting Summary linked to this article.

## Supplementary information

Supplementary Information

Reporting Summary

## Data Availability

The data that support the findings of this study are available from the corresponding author upon reasonable request.
