# Supplementary material for: The interaction of lipids and inflammatory markers predict negative symptom severity in patients with schizophrenia
Source: NPJ Schizophr. 2021 Oct 20;7:50. doi: 10.1038/s41537-021-00179-8 (PMC8528914; doi:10.1038/s41537-021-00179-8)
Supplement: Supplementary file 1 — Supplementary Information [file 41537_2021_179_MOESM1_ESM.pdf]

Supplementary Material

1. Additional information regarding the samples

Main Sample: Psychiatric diagnosis was confirmed by the Structured Clinical Interview for DSM-IV (SCID), Axis-I. Subjects were medically screened and excluded if they had a heart attack or heart failure within the last 60 days, neurological disease, head trauma, CNS infection, seizure disorder, HIV, autoimmune condition, or clinically significant hearing or visual impairment. Subjects were also excluded if they had intellectual disability, active substance abuse within three months of testing (as confirmed by SCID and urine toxicology on day of testing).

Replication Sample: Psychiatric diagnosis was confirmed by SCID and subjects with a diagnosis of schizophrenia, schizoaffective disorder, or psychosis not otherwise specified were included. Subjects were screened and excluded for history of immune disorders, HIV, hepatitis B or C, and current infection including urinary tract infection. Subjects were excluded if they used illicit drugs in the 30 days prior to study visit. Exclusion criteria also included use of anti-inflammatory drugs (e.g., non-steroidal anti-inflammatories, corticosteroids, other immunomodulatory agents). Subjects could not have taken an antibiotic within two weeks of study visit (based on self-report and review of the electronic medical record).

2. Rationale and Method for Combining Inflammatory Markers

TNF and IL6 were chosen from a panel of inflammatory markers due to their being two of the most common markers found to be elevated in patients with schizophrenia, including patients with chronic illness (Goldsmith et al., 2016). Additionally, we and others have previously published on their association with negative symptoms. We chose to investigate each marker individually as well as in combination. We took the sum of the Z-scores, a common method for examining the combination of multiple related inflammatory markers (Erdembileg et al., 2015; Felger et al., 2018; Haroon et al 2020; Hopkins et al 2012; Mehta et al 2020), and calculated the median split to determine a cut-off for low vs high groupings.

3. Analyses and Results from Replication Sample

In the replication sample, partial correlation analyses were run between TNF+IL6 group x cholesterol group and TNF+IL6 group x LDL group and total negative symptoms on the PANSS as well as the individual negative symptoms found to be significant in the primary analyses (blunted affect and emotional withdrawal) while controlling for age, sex, race, smoking, and BMI. There was a significant relationship between emotional withdrawal and the TNF+IL6 group x cholesterol group interaction ($r=0.280$, $p=0.030$) as well as with the TNF+IL6 group x LDL group interaction ($r=0.280$, $p=0.030$). Similarly, there was a significant correlation between blunted affect and TNF+IL6 group x cholesterol group interaction ($r=0.260$, $p=0.044$) and the TNF+IL6 group x LDL group interaction ($r=0.268$, $p=0.038$). There was not a significant correlation between total negative symptoms and TNF+IL6 group x cholesterol group interaction ($r=0.208$, $p=0.111$), though there was a trend for the correlation between the TNF+IL6 group x LDL group interaction ($r=0.230$, $p=0.077$).

Supplementary Table 1. Full Results

		beta	p-value
TNF Group x Total Cholesterol Group		0.456	0.491
IL6 Group x Total Cholesterol Group		1.359	0.053
TNF + IL6 group x Total Cholesterol Group		0.710	0.300
TNF Group x LDL Group		1.392	0.036
IL6 Group x LDL Group		0.378	0.584
TNF + IL6 Group x LDL Group		1.533	0.016
TNF Group x HDL Group		-0.104	0.562
IL6 Group x HDL Group		-0.119	0.492
TNF + IL6 Group x HDL Group		-0.077	0.664
TNF Group x TG Group		0.079	0.642
IL6 Group x TG Group		0.054	0.741
TNF + IL6 Group x TG Group		0.102	0.540
TNF Group x VLDL Group		0.055	0.749
IL6 Group x VLDL Group		0.005	0.975
TNF + IL6 Group x VLDL Group		0.062	0.720
TNF Group x TG:HDL Ratio Group		-0.011	0.952
IL6 Group x TG:HDL Ratio Group		-0.025	0.884
TNF + IL6 Group x TG:HDL Ratio Group		0.002	0.990

TNF: tumor necrosis factor; IL6: interleukin-6; LDL: low density lipoprotein; HDL: high density lipoprotein; TG: triglyceride; VLDL: very low density lipoprotein

Supplementary Table 2. Clinical Differences Between High and Low Inflammation Groups: Means and standard deviations were used to describe continuous variables (*) and independent sample t-tests were used to test differences. Percentages were used to describe categorical variables, chi-square tests were used to test differences for those with 2-categories (**), and one-way ANOVA tests were used for those with >2 categories (***).

	High Inflammation Group	Low Inflammation Group	T/x^2/F	p-value
Age*	50.15 years (10.13)	52.46 years (8.29)	0.899	0.373
Sex (% male)**	92.31%	96.15%	0.354	0.552
Race (% black)***	61.54%	88.46%	3.883	0.054
SGA Use***	80.77%	69.23%	0.592	0.445
Smoker**	46.15%	53.85%	0.308	0.579
BMI*	32.60 (8.68)	30.23 (3.85)	-1.273	0.211
PANSS Total Score*	62.88 (16.13)	58.35 (12.47)	-1.135	0.262
PANSS Positive Score*	16.23 (5.01)	15.42 (4.71)	-0.599	0.552
PANSS Negative Score*	17.31 (6.30)	15.85 (5.73)	-0.875	0.386
PANSS General Score*	29.35 (8.81)	27.08 (6.60)	-1.051	0.298

SGA: second generation antipsychotic; BMI: body mass index; PANSS: Positive and Negative Syndrome Scale
