# Supplementary material for: The interaction of lipids and inflammatory markers predict negative symptom severity in patients with schizophrenia
Source: NPJ Schizophr. 2021 Oct 20;7:50. doi: 10.1038/s41537-021-00179-8 (PMC8528914; doi:10.1038/s41537-021-00179-8)
Supplement: Supplementary file 2 — Reporting Summary [file 41537_2021_179_MOESM2_ESM.pdf]

nature portfolio

Corresponding author(s): David R. Goldsmith, MD MSc

Last updated by author(s): 7/17/2021

Reporting Summary

Nature Portfolio wishes to improve the reproducibility of the work that we publish. This form provides structure for consistency and transparency in reporting. For further information on Nature Portfolio policies, see our Editorial Policies and the Editorial Policy Checklist.

Statistics

For all statistical analyses, confirm that the following items are present in the figure legend, table legend, main text, or Methods section.

n/a	Confirmed	
☐	☒	The exact sample size (n) for each experimental group/condition, given as a discrete number and unit of measurement
☐	☒	A statement on whether measurements were taken from distinct samples or whether the same sample was measured repeatedly
☐	☒	The statistical test(s) used AND whether they are one- or two-sided<br>*Only common tests should be described solely by name; describe more complex techniques in the Methods section.*
☐	☒	A description of all covariates tested
☐	☒	A description of any assumptions or corrections, such as tests of normality and adjustment for multiple comparisons
☐	☒	A full description of the statistical parameters including central tendency (e.g. means) or other basic estimates (e.g. regression coefficient) AND variation (e.g. standard deviation) or associated estimates of uncertainty (e.g. confidence intervals)
☐	☒	For null hypothesis testing, the test statistic (e.g. F, t, r) with confidence intervals, effect sizes, degrees of freedom and P value noted<br>*Give P values as exact values whenever suitable.*
☒	☐	For Bayesian analysis, information on the choice of priors and Markov chain Monte Carlo settings
☒	☐	For hierarchical and complex designs, identification of the appropriate level for tests and full reporting of outcomes
☒	☐	Estimates of effect sizes (e.g. Cohen's d, Pearson's r), indicating how they were calculated

Our web collection on statistics for biologists contains articles on many of the points above.

Software and code

Policy information about availability of computer code

Data collection	no software used
Data analysis	SPSS 27

For manuscripts utilizing custom algorithms or software that are central to the research but not yet described in published literature, software must be made available to editors and reviewers. We strongly encourage code deposition in a community repository (e.g. GitHub). See the Nature Portfolio guidelines for submitting code & software for further information.

Data

Policy information about availability of data

All manuscripts must include a data availability statement. This statement should provide the following information, where applicable:

- Accession codes, unique identifiers, or web links for publicly available datasets
- A description of any restrictions on data availability
- For clinical datasets or third party data, please ensure that the statement adheres to our policy

The data that supports the findings of this study are available from the corresponding author upon reasonable request.

Field-specific reporting

Please select the one below that is the best fit for your research. If you are not sure, read the appropriate sections before making your selection.

☐ Life sciences ☒ Behavioural & social sciences ☐ Ecological, evolutionary & environmental sciences

For a reference copy of the document with all sections, see nature.com/documents/nr-reporting-summary-flat.pdf

Behavioural & social sciences study design

All studies must disclose on these points even when the disclosure is negative.

Study description	Quantitative Cross Sectional Data
Research sample	Patients with schizophrenia
Sampling strategy	Patients were recruited to participate in the study from the Atlanta Veteran Affairs Health System and sample size was based on the number of samples run for the inflammatory marker assays
Data collection	Negative Symptoms were assessed using the Positive and Negative Syndrome Scale by trained raters at study visit. Blood samples for immune markers were obtained in chilled EDTA-coated tubes and spun at 2000g for 15 minutes at 4 degrees C, and plasma was collected and stored at -80 degrees C for later batched analysis. Blood for lipids were obtained from the electronic medical record and recorded from the closest date to the study visit. Time between lipid blood draw and study visit (days) was included as a covariate in the analyses.
Timing	Data collection started on 3/1/2016 and ended on 10/1/2017
Data exclusions	No data was excluded, though only subjects who had samples to run inflammatory markers were included.
Non-participation	Of 98 subjects who were consented, 17 screened out due to the following reasons: age outside the range, positive urine toxicology, failed hearing test, and end state renal disease.
Randomization	There was no randomization.

Reporting for specific materials, systems and methods

We require information from authors about some types of materials, experimental systems and methods used in many studies. Here, indicate whether each material, system or method listed is relevant to your study. If you are not sure if a list item applies to your research, read the appropriate section before selecting a response.

Materials & experimental systems

n/a	Involved in the study	
☒	☐	Antibodies
☒	☐	Eukaryotic cell lines
☒	☐	Palaeontology and archaeology
☒	☐	Animals and other organisms
☐	☒	Human research participants
☒	☐	Clinical data
☒	☐	Dual use research of concern

Methods

n/a	Involved in the study	
☒	☐	ChIP-seq
☒	☐	Flow cytometry
☒	☐	MRI-based neuroimaging

Human research participants

Policy information about studies involving human research participants

Population characteristics	Males and females with schizophrenia were recruited for the study. There were a number of exclusion criteria described in the manuscript.
Recruitment	Subjects were recruited from the Atlanta Veterans Affairs Medical Center.
Ethics oversight	Emory University Institutional Review Board and the Atlanta VAMC Research and Development Committee.

Note that full information on the approval of the study protocol must also be provided in the manuscript.
